# Techniques and Outcomes of the No-Touch Vein Conduit as a Y-Composite Graft

**DOI:** 10.21470/1678-9741-2022-0119

**Published:** 2022

**Authors:** Ki-Bong Kim, Seong Wook Hwang, Min-Seok Kim

**Affiliations:** 1 Cardiovascular Center, Myongji Hospital, Goyang-si, Gyeonggi-do, Republic of Korea

**Keywords:** Coronary Artery Bypass, Venous Grafts, Saphenous Vein, Transplants, Mammary Arteries.

## Abstract

Although the saphenous vein is a widely used conduit for coronary artery bypass grafting, revascularization using the saphenous vein as an aortocoronary bypass graft has shown disadvantages of lower long-term graft patency rates and subsequently worse clinical outcomes, compared with revascularization using the internal thoracic artery. Of the various efforts to overcome the limitations of vein conduit that are resulting from structural and functional differences from arterial conduit, recent technical improvement in no-touch vein composite graft construction and outcomes of revascularization using no-touch vein composite grafts based on the left internal thoracic artery will be discussed in this topic.

**Table:** 

Abbreviations, Acronyms & Symbols	
CABG	= Coronary artery bypass grafting
ITA	= Internal thoracic artery
MACCE	= Major adverse cardiac and cerebrovascular events
RITA	= Right internal thoracic artery
SAVE RITA	= SAphenous VEin *versus* Right Internal Thoracic Artery as a Y-Composite Graft
SV	= Saphenous vein

## INTRODUCTION

Revascularization using an *in situ* left internal thoracic artery (ITA)-based composite graft has advantages of avoiding aortic manipulation and allowing efficient conduit utilization. Complete revascularization using a composite graft constructed with arterial conduits, including right ITA (RITA), radial artery, and right gastroepiploic artery, has been demonstrated to be a safe and efficient method^[^1^-^4^]^; however, previous studies describing the use of the saphenous vein (SV) as a composite graft have produced conflicting results^[^5^-^8^]^. Herein, techniques and outcomes of the no-touch SV conduit as a Y-composite graft based on the *in situ* left ITA will be discussed.

### Techniques of No-Touch Vein Composite Graft Construction and Strategies of Revascularization

The harvesting technique of the no-touch SV and strategies of coronary artery bypass grafting (CABG) have been previously described^[^9^,^10^]^. SV harvest was initiated after systemic heparinization therapy during harvesting of the left ITA and was performed using an open technique. Patients were given an initial dose of heparin (1.5 mg/kg) and periodic supplemental doses to maintain an activated clotting time of ≥ 300 seconds. The SV harvest was performed using the no-touch technique without or with surrounding pedicle tissue, in which the manipulation and tension of the SV were minimized during harvesting, and manual intraluminal dilatation was avoided. In the no-touch SV harvest without pedicle tissue, the vein was gently separated from the bed using scissors, leaving perivascular scanty adipose tissue in place. In the no-touch SV harvest with pedicle tissue, the SV pedicle was harvested along with an approximately 3- to 5-mm wide margin of adjacent adipose tissues on both sides of the SV and thin layers of adherent connective tissues posteriorly.

Immediately after the harvest and with no pharmacologic treatment, the reversed SV was anastomosed in a parallel fashion to the posterior aspect of the left ITA to construct a Y-composite graft. We always performed the Y anastomosis first, before constructing distal anastomoses, because it was possible to perfuse ischemic myocardium after each distal anastomosis during off-pump CABG. After construction of the Y-composite graft, the left coronary artery territory commonly was revascularized first by using the left ITA while the distal end of the SV conduit was clamped with an atraumatic bulldog clamp and left to be dilated spontaneously by the native flow and pressure of the left ITA.

The valves of the spontaneously dilated SV were then destroyed by inserting a 2-mm round-edge vessel dilator, which was much smaller than the diameter of the dilated SV and might minimize endothelial damage of the SV trunk into the reversed SV lumen. We assumed that leaving the SV valve intact might cause blood stagnation between the sequential distal anastomoses in the event of flow competition and result in early graft failure. Destruction of the valve may cause endothelial injury in the SV graft; however, we believe a gain with valve destruction is greater than losses without valve destruction^[^11^]^. The left circumflex coronary artery territory was then revascularized using the SV as a composite conduit, followed by the right coronary artery territory. A sequential anastomotic technique used each side arm of the Y-composite graft for complete revascularization when more than two coronary arterial anastomoses were needed. Longitudinal, perpendicular, or oblique sequential side-to-side anastomoses were performed for construction of sequential anastomoses to permit efficient use of the conduit. All the anastomoses were performed with an 8-0 polypropylene continuous suture, using a high-power magnification loupe (× 4.5 magnification). Transit-time flow measurement (Medi-Stim AS, Oslo, Norway) was used to verify the anastomosis status after each anastomosis was performed and just before pericardial closure. All patients received preoperative aspirin therapy (100 mg daily) until the day of surgery and resumed it postoperatively as soon as possible (usually 1 day). Clopidogrel (75 mg daily) was added simultaneously to the aspirin therapy for one year postoperatively. If the patient had a high blood level of low-density lipoprotein cholesterol (> 70 mg/dL), statin therapy was initiated and maintained postoperatively.

### Outcomes of Revascularization Using the No-Touch Vein Composite Grafts

**Fig. 1:**
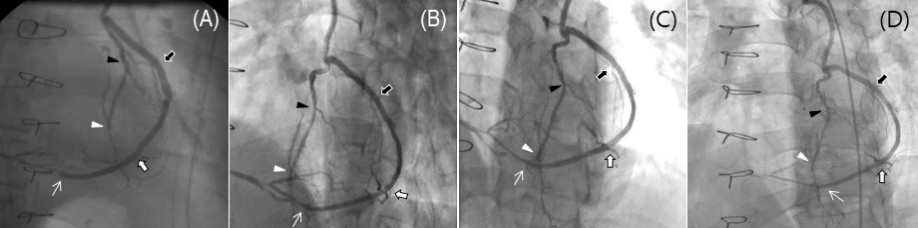
Patent no-touch saphenous vein (SV) Y-composite grafts based on the in situ left internal thoracic artery (ITA) at (A) early postoperative, (B) 1-year, (C) 5-year, and (D) 10-year angiographies in a 54-year-old male patient enrolled in the SAVE RITA trial. The in situ left ITA was anastomosed to the second diagonal (black arrowheads) and left anterior descending coronary arteries (white arrowheads), and the SV was anastomosed to the first diagonal (black arrows) and distal obtuse marginal (white arrows) and right posterolateral coronary arteries (white thin arrows) using a sequential anastomotic technique. SAVE RITA=SAphenous VEin versus Right Internal Thoracic Artery as a Y-Composite Graft

**Fig. 2:**
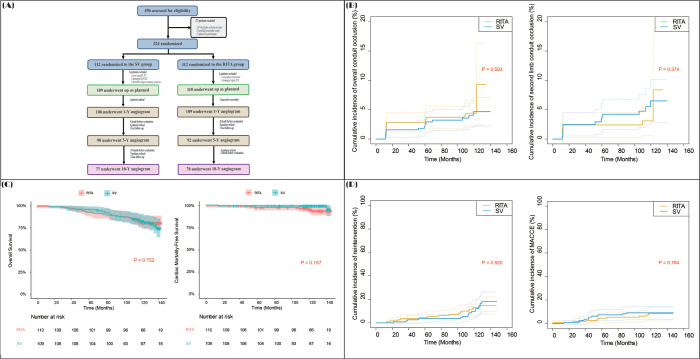
Long-term angiographic and clinical results of the SAVE RITA trial. (A) Summary flow diagram of patients. Comparison of (B) cumulative incidence of the overall conduit and second-limb conduit occlusion, (C) overall survival and cardiac mortality-free survival, (D) cumulative incidence of reintervention and major adverse cardiac and cerebrovascular events (MACCE) between the saphenous vein (SV) and right internal thoracic artery (RITA) groups. ITA=internal thoracic artery; SAVE RITA=SAphenous VEin versus Right Internal Thoracic Artery as a Y-Composite Graft

Recent studies demonstrated that long-term clinical outcomes of bilateral ITA composite grafting were comparable to those of bilateral ITA *in situ* grafting and also demonstrated that long-term clinical outcomes of other arterial composite grafting using radial or right gastroepiploic artery were comparable to those of bilateral ITA composite grafting^[^1^-^4^]^. In contrast, the use of the SV as a composite graft has produced conflicting clinical results. One study recommended against the use of an SV composite graft because it could steal flow from the left ITA conduit and lead to suboptimal short-term ITA patency results (perfect patency of ITA grafts, 76% at a mean 2.5 years)^[^5^]^. In contrast, other studies demonstrated comparable hemodynamic characteristics and patency results between the SV and arterial composite grafts. One hemodynamic study measured the pressure gradient and fractional flow reserve of composite grafts made with RITA or SV based on the *in situ* left ITA and showed that hemodynamics of RITA and SV composite grafts were similar in terms of pressure gradients at baseline and hyperemia, and fractional flow reserve^[^6^]^. Other studies, in which the no-touch SV was harvested without surrounding pedicle tissue, demonstrated comparable long-term clinical and patency results between the SV and arterial composite grafts^[^7^,^8^]^. A retrospective study comparing results of CABG using no-touch SV composite grafts and arterial composite grafts demonstrated that the patency rates of the SV and arterial composite grafts were similar in propensity score-matched groups (SV *vs.* RITA; 95.9% [71 of 74 distal anastomoses] *vs.* 87.3% [96 of 110], *P*=0.702) 10 years after surgery^[^7^]^. Another recent extended study of the SAVE RITA (SAphenous VEin *versus* Right Internal Thoracic Artery as a Y-Composite Graft) trial demonstrated that the no-touch SV composite grafts were comparable to the RITA composite grafts in terms of 10-year conduit occlusion rates and long-term clinical outcomes^[^8^]^. The 10-year occlusion rate of SV second limb conduits in the SV group was comparable with RITA second limb conduits of the RITA group (6.9% *vs.* 3.4%; *P*=0.213). There were no significant intergroup differences in the overall survival rates, freedom from cardiac death rates, and cumulative incidence of reintervention and major adverse cardiac and cerebrovascular events (Figures 1 and 2).

Theoretical advantages of SV composite grafts based on the *in situ* ITA over an aortocoronary bypass graft include: (1) the SV conduit anastomosed to the ITA is exposed to less circulatory stress than a conduit anastomosed to the ascending aorta; (2) the SV composite graft is continuously exposed to endothelium-protective substances such as nitric oxide released from the ITA. Complete revascularization using an SV composite graft based on the *in situ* ITA also has advantages such as avoiding aortic manipulation and allowing efficient use of bypass conduits. The length of SV needed to reach the target vessel is shorter than that of an aortocoronary bypass graft when using an SV composite graft with a sequential anastomosis technique. The SV from a lower or upper leg is sufficient for complete revascularization in most patients with multivessel coronary artery disease^[^12^]^. An additional observational study demonstrated that the no-touch SV conduits with surrounding pedicle tissue further improved the early and one-year patency of SV composite grafts compared with those of no-touch SV composite grafts without surrounding pedicle tissue, which might result from improving patency of the no-touch SV conduits by maintaining pulsatility of the cushioned graft^[^10^]^.

## CONCLUSION

Recent advances in no-touch SV harvesting techniques and grafting strategy of SV as a composite graft based on the *in situ* ITA may improve long-term patency of the SV conduits in CABG. Preserved SV endothelial wall structures and exposure to substances of the *in situ* ITA may lead to favorable negative remodeling of the SV. With pre-existing advantages of the SV conduit, such as ease of access, enough length, and short-operation time by simultaneous harvesting with the ITA, the improved patency of the no-touch SV composite grafts will make this conduit more valuable for CABG.

**Table:** 

Authors’ Roles & Responsibilities	
KBK	Drafting the work or revising it critically for important intellectual content; final approval of the version to be published
SWH	Drafting the work or revising it critically for important intellectual content; final approval of the version to be published
MSK	Drafting the work or revising it critically for important intellectual content; final approval of the version to be published
